# Deciphering MMRN1 diagnostic and therapeutic implications in the substantia nigra of Parkinson’s disease patients via integrative bioinformatic analysis and multi-omics studies

**DOI:** 10.3389/fnagi.2026.1761327

**Published:** 2026-06-18

**Authors:** Yu Ning, Wei Gao, Yan Gao

**Affiliations:** 1 General Surgery, Affiliated Hospital of Beihua University, Jilin City, Jilin, China; 2 Ophthalmology Department, Jilin People's Hospital, Jilin City, Jilin, China; 3 Neurology Department, Affiliated Hospital of Beihua University, Jilin City, Jilin, China

**Keywords:** drug repurposing, machine learning, multi-omics, Parkinson’s disease, substantia nigra, summary-data-based Mendelian randomization

## Abstract

**Background:**

Parkinson’s disease (PD) is a neurodegenerative disorder characterized by the loss of dopaminergic neurons in the substantia nigra (SN), primarily due to *α*-synuclein aggregation, posing a major health threat to the elderly population. Current treatment options remain limited, necessitating the discovery of novel diagnostic and therapeutic targets.

**Objective:**

This study aimed to identify a novel diagnostic and druggable target in the SN of PD patients.

**Methods:**

We first nominated PD risk-associated differentially expressed genes (DEGs) in the SN bulk profile (GSE7621) of PD patients using Limma, weighted gene co-expression network analysis (WGCNA), and summary-data-based Mendelian randomization (SMR). Next, three machine learning algorithms [random forest (RF), least absolute shrinkage and selection operator LASSO, and support vector machine (SVM)] were performed for the identification of central pathogenic factors among the risk DEGs in the training PD patient SN bulk profile (integrated GSE20163 and GSE20164). In addition, the diagnostic performance of the identified central pathogenic factor for PD was evaluated in GSE7621, integrated GSE20163 and GSE20164, and independent PD patient SN bulk profiles (GSE140231). In addition, the molecular and immune patterns of the central pathogenic factor were assessed in SN single-cell data from PD patients (GSE7621) were estimated by a cutting-edge analytical framework in temporal and spatial manners. Furthermore, network-based drug screening and molecular docking were subsequently performed to identify potential therapeutic agents targeting the central pathogenic factors. Finally, *in vitro* assays estimated the expression patterns of the central pathogenic factor.

**Results:**

Multimerin 1 (MMRN1) was identified as an upregulated pathogenic factor predominantly expressed in neurons. Quercetin was highlighted as a promising repurposed drug candidate targeting MMRN1.

**Conclusion:**

This study illustrated a novel diagnostic and therapeutic target for PD, which provides novel clues into clinical applications of PD patients.

## 1 Introduction

Parkinson’s disease (PD) is a progressive neurodegenerative disorder (NDD) primarily affecting the elderly population, with an estimated global prevalence of approximately 10 million cases (Bloem et al., 2021). The disease is characterized by the degeneration of dopaminergic neurons in the substantia nigra (SN), leading to a significant reduction in dopamine levels, which is crucial for motor function (Tolosa et al., 2021). Epidemiologically, PD presents a higher incidence and mortality rate among older adults, highlighting the urgent need for effective therapeutic strategies to combat its progression (Elsworth, 2020).

At the molecular level, PD pathogenesis is primarily involved in the accumulation of misfolded alpha-synuclein (*α*-Syn), which aggregates to form Lewy bodies (Tanner and Ostrem, 2024). Indeed, this process is associated with neurodegenerative changes, including oxidative stress and neuroinflammation, which further contribute to the death of dopaminergic neurons (Hayes, 2019). Research has established that signaling pathways, such as apoptosis and oxidative stress pathways, play critical roles in neuronal cell death and the overall pathophysiology of PD (Warnecke et al., 2023; Schneider and Alcalay, 2020). Due to the complexity of PD etiology and the multifaceted nature of its progression, current therapeutic options are limited and predominantly symptomatic, underscoring the urgent need for innovative treatment strategies that target these underlying molecular mechanisms to halt or reverse disease progression (Reich and Savitt, 2019).

In recent years, artificial intelligence (AI) algorithms, such as machine learning and deep learning, with integrative bioinformatic analysis approaches, have boosted the screening of NDDs, including decision-making, diagnosis, health monitoring, and therapeutic strategies development (Tăuţan et al., 2021). In brief, research focused on AI with bioinformation can bring concrete benefits for neurodegenerative patients and holds a broad market in the medical application area.

**Figure 1:**
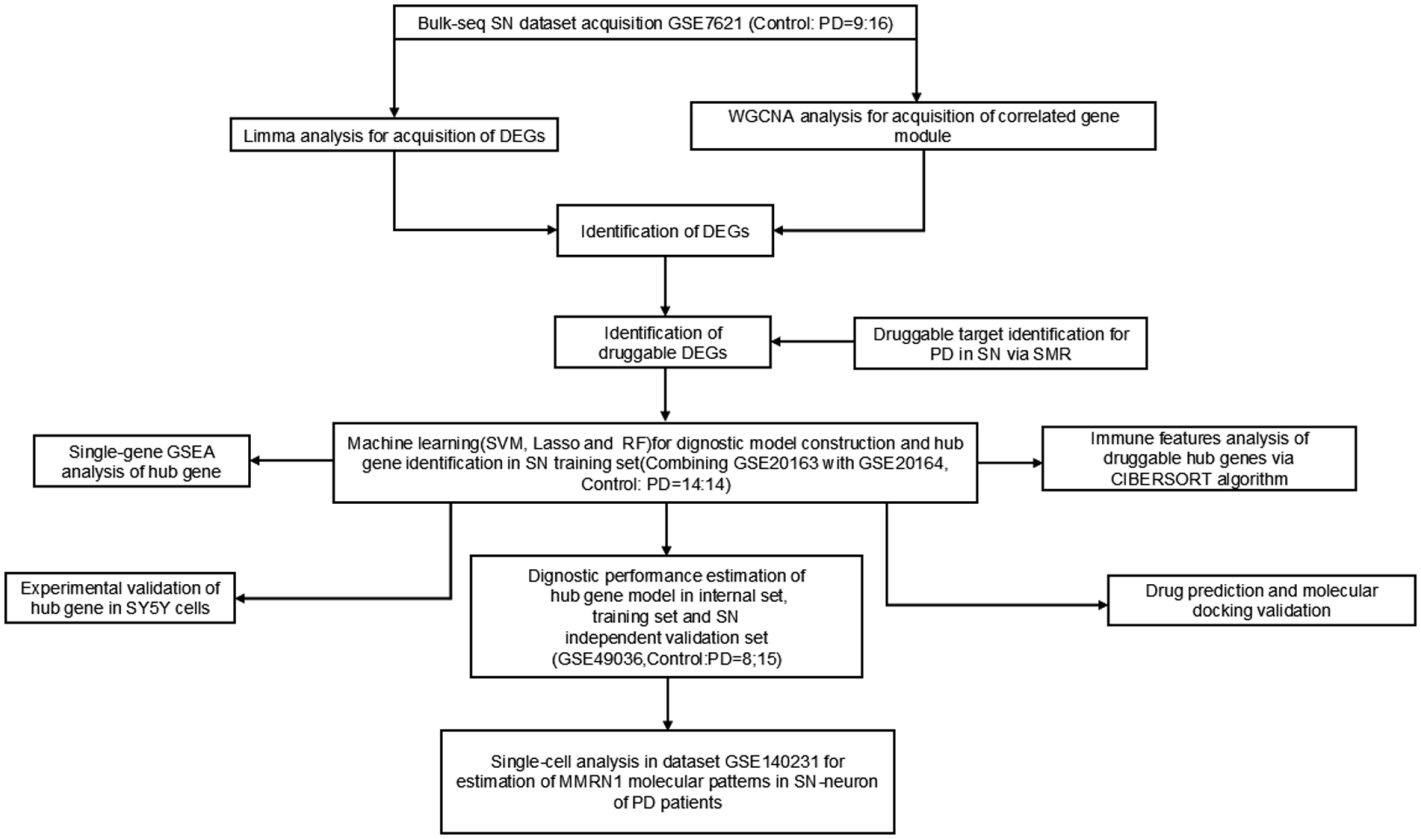
Workflow of this study.

Overall, we performed integrative and multi-omics studies to discover the molecular mechanism and therapeutic strategies for the treatment of PD. First, the public SN bulk-seq dataset (GSE7621) of PD patients was analyzed for the identification of co-expression differentially expressed genes (DEGs) using Limma and WGCNA. Next, summary-data-based Mendelian randomization (SMR) was used for the druggable target discovery for PD patients in the SN. By combining the DEGs with SMR results, we identified druggable DEGs in the SN for PD patients. Subsequently, by integrating the GSE20163 and GSE20164 datasets (SN bulk-seq datasets of PD patients) with three machine learning algorithms (RF, LASSO, and SVM), we identified Multimerin 1 (MMRN1) that can be considered as a hub druggable gene involved in PD pathogenesis. Next, the independent SN validation set of PD patients (GSE49036) with the aforementioned datasets was used for MMRN1 diagnostic performance evaluation of PD patients via receiver operating characteristic (ROC), precision–recall (PR), decision curve analysis (DCA), nomogram, and calibration analysis. The results indicated that MMRN1 can be considered as a diagnostic marker for PD. In addition, a single-cell transcriptomic dataset (GSE140231) illustrated that MMRN1 is primarily distributed in neurons and is associated with neuroinflammation and blood–brain barrier maintenance. Furthermore, network-based drug efficacy screening and molecular docking illustrated quercetin’s therapeutic potential in the treatment of PD. Finally, an SY5Y-oriented *in vitro* study revealed that the expression of MMRN1 was upregulated. Our study first reported the diagnostic role of MMRN1 in PD and offers novel clinical therapeutic strategies. We describe the workflow of this study in Figure 1.

## 2 Materials and methods

### 2.1 Bulk-seq data source

Initially, we obtained four SN microarray datasets of PD patients from the Gene Expression Omnibus (GEO) database using the GEOquery package in R (Davis and Meltzer, 2007). The datasets included GSE7621 (including 8 control samples and 16 PD patient samples), GSE20163 (including 9 control samples and 8 PD patient samples), GSE20164 (including 5 control samples and 6 PD patient samples), and GSE49036 (including 8 control samples and 15 PD patient samples). We integrated GSE20163 and GSE20164 and then removed the batch effect through the sva package in R (Leek et al., 2012). All three of these datasets were normalized using the preprocessCore package in R (Yang and Mei, 2015). GSE7621 was designated as the internal set. The integrated GSE20163 and GSE20164 dataset was designated as the training set. The GSE49036 dataset was designated as the independent validation set.

### 2.2 Identification of DEGs and WGCNA

Differential expression analysis was conducted on the internal dataset using the Limma package within R software (Ritchie et al., 2015). Differentially expressed genes (DEGs) were identified using the criteria of | log2FC | > 0.5 and a *p*-value of <0.05, and their results were illustrated through volcano plots and heatmaps created using the ggplot2 and complexheatmap packages (Cao et al., 2023; Gu, 2022). Furthermore, the WGCNA package in R was used to explore the relationships between genes and phenotypes by constructing a gene co-expression network for the internal dataset (Langfelder and Horvath, 2008). Initially, we eliminated 50% of the genes exhibiting the lowest median absolute deviation (MAD). Subsequently, we computed Pearson’s correlation matrices for all conceivable gene pair comparisons and established a weighted adjacency matrix using the average linkage method, alongside a weighted correlation coefficient. The “soft” thresholding power (*β*) was then applied to determine the adjacency, which was subsequently transformed into a topological overlap matrix (TOM). To categorize genes with similar expression profiles into modules, average linkage hierarchical clustering was performed based on the dissimilarity measure derived from the TOM, ensuring that each module contained at least 50 genes. Ultimately, we assessed the dissimilarity of module eigengenes, set a cutoff for the module dendrogram, and merged several modules. The WGCNA led to the identification of significant modules associated with Parkinson’s disease (PD), culminating in the creation of a visualized eigengene network.

### 2.3 SMR analysis

SMR is a statistical methodology that combines genome-wide association studies (GWAS) with expression quantitative trait loci (eQTL) analyses to investigate the intricate relationships between gene expression and various complex traits of interest (Zhu et al., 2016). Subsequently, we accessed GWAS data (ebi-a-GCST90018894) related to PD from the IEU Open GWAS database using the TwoSampleMR package in R software (Hemani et al., 2018). Notably, all outcome data were sourced from individuals of European ancestry. In this context, we conducted a two-sample Mendelian randomization analysis using the TwoSampleMR package in R, where the exposure variable was a druggable protein and the outcome of interest was PD. To uphold the validity of the independence assumption, stringent criteria were applied during the selection of single-nucleotide polymorphisms (SNPs). This process entailed excluding SNPs associated with the major histocompatibility complex (MHC) region and addressing linkage disequilibrium to minimize the potential impact of confounding biases. Moreover, the Wald ratio method was used to evaluate the outcomes of Mendelian randomization, considering exposure from a single SNP (Li et al., 2025). If the exposure involved two or more SNPs, the inverse variance weighting (IVW) method was used to assess the Mendelian randomization (MR) results. Finally, the TwoSampleMR package in R was also leveraged to perform tests for heterogeneity and pleiotropy, followed by the implementation of the Steiger directional test to confirm the reliability of the causality direction (Li et al., 2025). We next combined the results of Limma, WGCNA, and SMR for the identification of druggable DEGs.

### 2.4 Machine learning algorithms and diagnostic model construction

A LASSO logistic regression analysis constitutes an advanced data mining technique that employs an L1 penalty (lambda) to effectively reduce the coefficients of less significant variables to zero (Zhang et al., 2021). This methodology allows for the identification of pivotal variables, thereby aiding in the formulation of an optimal classification model. The SVM-RFE approach is a supervised machine learning technique that seeks to identify the most essential core genes by systematically removing feature vectors generated by the support vector machine (Lin et al., 2017). RF analysis is a machine learning method based on decision trees that concentrates on assessing the significance of variables through scoring the importance of each variable (Qu et al., 2022). By integrating these three machine learning algorithms with a training dataset, we identified the hub variable implicated in the pathogenesis of PD. Subsequently, the molecular function of the hub variable was evaluated within the training set by single-gene GSEA analysis, in accordance with the hallmark gene set sourced from the MSIGDB database (Subramanian et al., 2005). Furthermore, the immune characteristics of the hub variable were estimated using the CIBERSORT algorithm (Li et al., 2022). The expression and diagnostic value of the hub variable were further validated across internal, training, and independent datasets. The diagnostic performance of the hub variable was determined through the use of the receiver operating characteristic (ROC) curves, precision–recall (PR) analysis, decision curve analysis (DCA), nomograms, and calibration by using the pROC, rms, and rmda packages within R software.

### 2.5 Single-cell transcriptomic analysis

Single-cell RNA sequencing data from the GSE140231 dataset (comprising 7 SN samples from PD patients) were processed using the Seurat package in R (Zhou et al., 2026). Low-quality cells were filtered out using thresholds of fewer than 200 or more than 6,000 unique features, or >20% mitochondrial gene content (Zhou et al., 2026). Following normalization and scaling, dimensionality reduction was performed using UMAP and t-SNE analysis (Zhou et al., 2026). The cells were clustered using the FindNeighbors and FindClusters functions (resolution = 0.5) and annotated by the scMayoMap package in R using the Human Primary Cell Atlas (HPCA) as a reference (Yang et al., 2023). To investigate intercellular signaling between annotated cells, the CellPhoneDB package in R was used by ligand–receptor patterns (Efremova et al., 2020). Metabolic heterogeneity across cell types was assessed using the scMetabolism package in R (Argüello et al., 2020). The expression pattern of the hub gene was visualized in dot embeddings. A virtual knockout (KO) of the hub gene within the targeted cell cluster was simulated using the scTenifoldKnk package in R with a *p*-value of <0.05 (Osorio et al., 2022). The top differentially expressed genes from this simulation were analyzed for functional enrichment. Kyoto Encyclopedia of Genes and Genomes (KEGG) and Gene Ontology (GO) analyses were conducted via the clusterProfiler package in R, with KEGG and GO gene sets obtained from the MSigDB database (Yu et al., 2012). Finally, pseudotime trajectory analysis of astrocytes was performed using the Monocle2 package in R (Huang et al., 2024).

### 2.6 Cell lines and culture conditions

Authenticated human dopaminergic (DA) neuron SH-SY5Y cells were sourced from the Shanghai Institute of Cell Biology (Shanghai, China) and maintained in Dulbecco’s Modified Eagle Medium (DMEM) enriched with 10% fetal bovine serum (FBS) and 1% penicillin–streptomycin. The cultures were incubated at 37 °C in a humidified environment with 5% CO₂. The culture medium was replaced every 2 to 3 days, and cell passages were conducted once the confluence reached approximately 80%. To imitate neuronal damage, SH-SY5Y cells were subjected to ultrapure MPP + (200 mM; #D048, Sigma-Aldrich, St. Louis, MO, United States) for 24 h at 37 °C. MPP + -treated SH-SY5Y cells were used for simulating PD, while untreated SH-SY5Y cells served as the normal control group.

### 2.7 RNA extraction and quantitative real-time PCR (qRT-PCR)

Total RNA was extracted using TRIzol reagent (TaKaRa, Beijing, China), and its concentration, purity, and integrity were subsequently evaluated using a NanoDrop spectrophotometer (Thermo Scientific, Waltham, MA, United States). Reverse transcription was performed on 1 μg of total RNA utilizing HiScript II Q RT SuperMix for quantitative polymerase chain reaction (qPCR) (+gDNA wiper), in combination with a gDNA eraser (Vazyme, Shanghai, China). The concentration, purity, and integrity of the synthesized cDNA were reassessed using the NanoDrop spectrophotometer (Thermo Scientific, Waltham, MA, United States). Quantitative reverse transcription PCR (qRT-PCR) was conducted using SYBR Green MasterMix (11203ES50, YEASEN, Shanghai, China) and analyzed using StepOne Software v.2.3 (Applied Biosystems, Carlsbad, CA, United States), incorporating a total of 40 amplification cycles across three biological replicates. Data analysis was executed employing the ∆∆Ct (cycle threshold) method, with normalization against the expression levels of the housekeeping gene, GAPDH. The sequences of the primers designed for the target gene were as follows (5′-3′) (Zhang et al., 2026):

MMRN1:

F: GGCATTGGGCTTAACAACAGT.

R: CGACATGACCCGAGTGGTT.

GAPDH:

F: GAGAAGGCTGGGGCTCATTT.

R: ATGACGAACATGGGGGCATC.

### 2.8 Drug enrichment and molecular docking analysis

Network-based *in silico* drug efficacy screening is a powerful tool for understanding the network-based origins of diseases and for introducing a drug–disease proximity measure that quantifies the interplay between drug targets and diseases (Guney et al., 2016). By correcting for the known biases of the interactome, proximity helps us uncover the therapeutic effect of drugs, as well as distinguish palliative from effective treatments (Guney et al., 2016). Using this approach, we enriched a therapeutic agent targeting a hub gene for the treatment of PD, pending molecular docking validation. Subsequently, molecular docking analyses were conducted to assess the binding interactions between the most effective drug and the central gene protein (Wang et al., 2022). The three-dimensional (3D) configurations of the active component (Compound CID: 5280343 for quercetin) and the hub target proteins (ID: AF-Q13201-F1) were obtained from the PubChem and Alphafold2 databases. Molecular docking was performed to validate the binding interaction between the candidate drug and the targeted protein. Semi-flexible docking was conducted using AutoDock Vina (version 1.2.0), where the protein was kept rigid while all rotatable bonds in the ligand were allowed to move freely. A docking grid box encompassing the entire targeted protein was defined. The exhaustiveness parameter was set to 24 to ensure thorough conformational sampling. The binding pose with the lowest calculated binding affinity (ΔG in kcal/mol) was selected and visualized using PyMOL (version 2.5.0) to analyze key hydrogen bonds and hydrophobic interactions (Wang et al., 2022).

### 2.9 Statistical analysis

All bioinformatic analyses were based on R software (version 4.2.2). Group comparisons were performed using Student’s *t*-test or the Mann–Whitney *U*-test for continuous variables. For multiple group comparisons, a one-way analysis of variance (ANOVA) or the Kruskal–Wallis test was used, followed by appropriate *post-hoc* tests. Correlation analyses used Pearson’s or Spearman’s methods. All statistical tests were two-sided, and a *p*-value of <0.05 and an FDR-value of <0.05 were considered significant.

## 3 Results

### 3.1 Identification of DEGs in SN for PD patients

**Figure 2:**
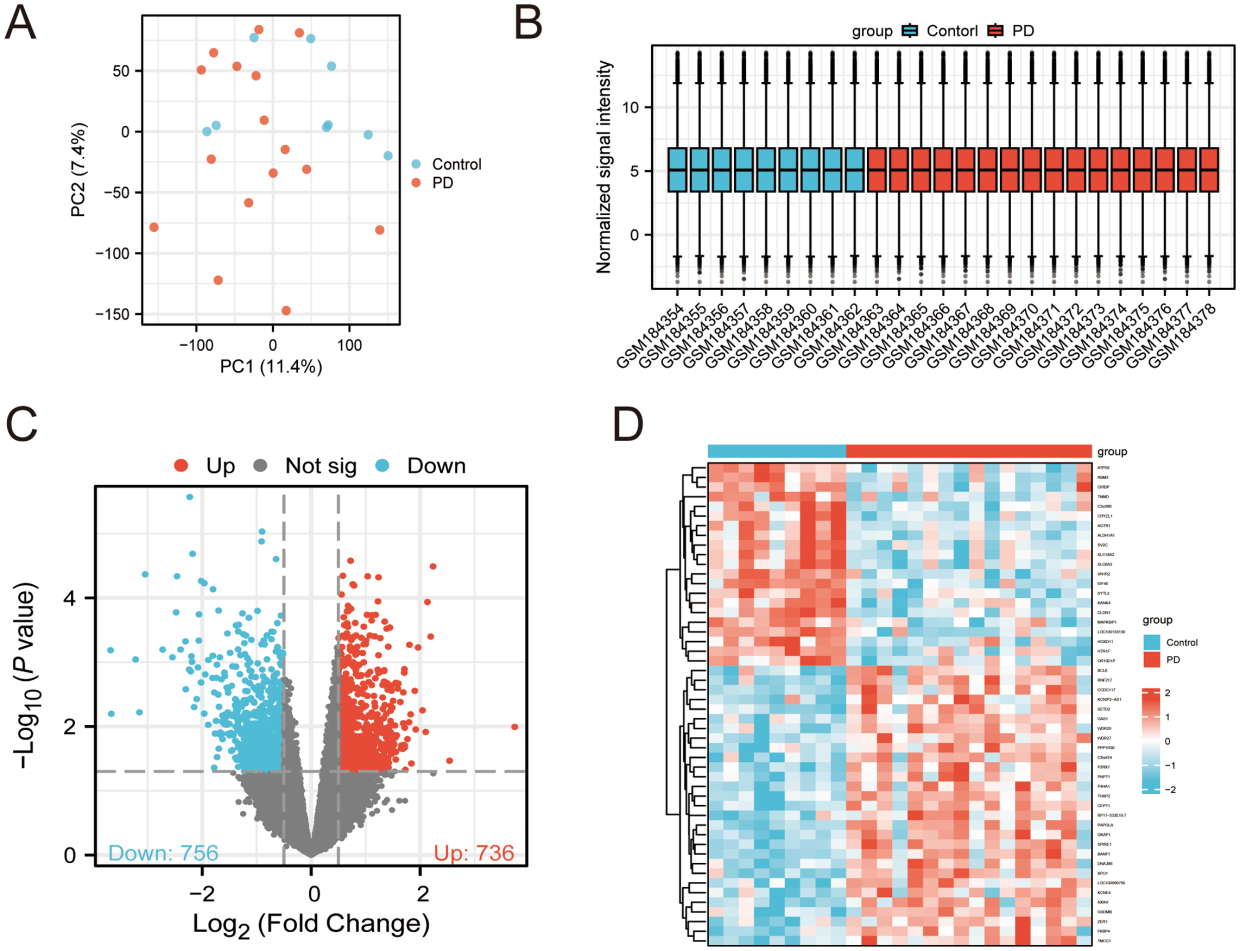
Identification of DEGs for PD patients. **(A)** PCA plot of dimensional reduction results in the internal set. **(B)** Box plot of normalization results in the internal set. **(C)** Volcano map illustration of DEGs in the internal set. **(D)** Distinct DEGs heatmap illustration in the internal set.

In the internal set, to identify candidate genes associated with PD, we first performed quality control and normalization. Principal component analysis (PCA) revealed a clear separation trend between PD patients and healthy controls (Figure 2A). Boxplots after normalization confirmed consistent expression distributions across samples (Figure 2B). A total of 1,492 DEGs were identified, including 736 upregulated and 756 downregulated genes (Figure 2C). Heatmap analysis further illustrated distinct expression profiles of DEGs between PD patients and controls (Figure 2D).

### 3.2 Identification of correlation gene modules in the SN for PD patients

**Figure 3:**
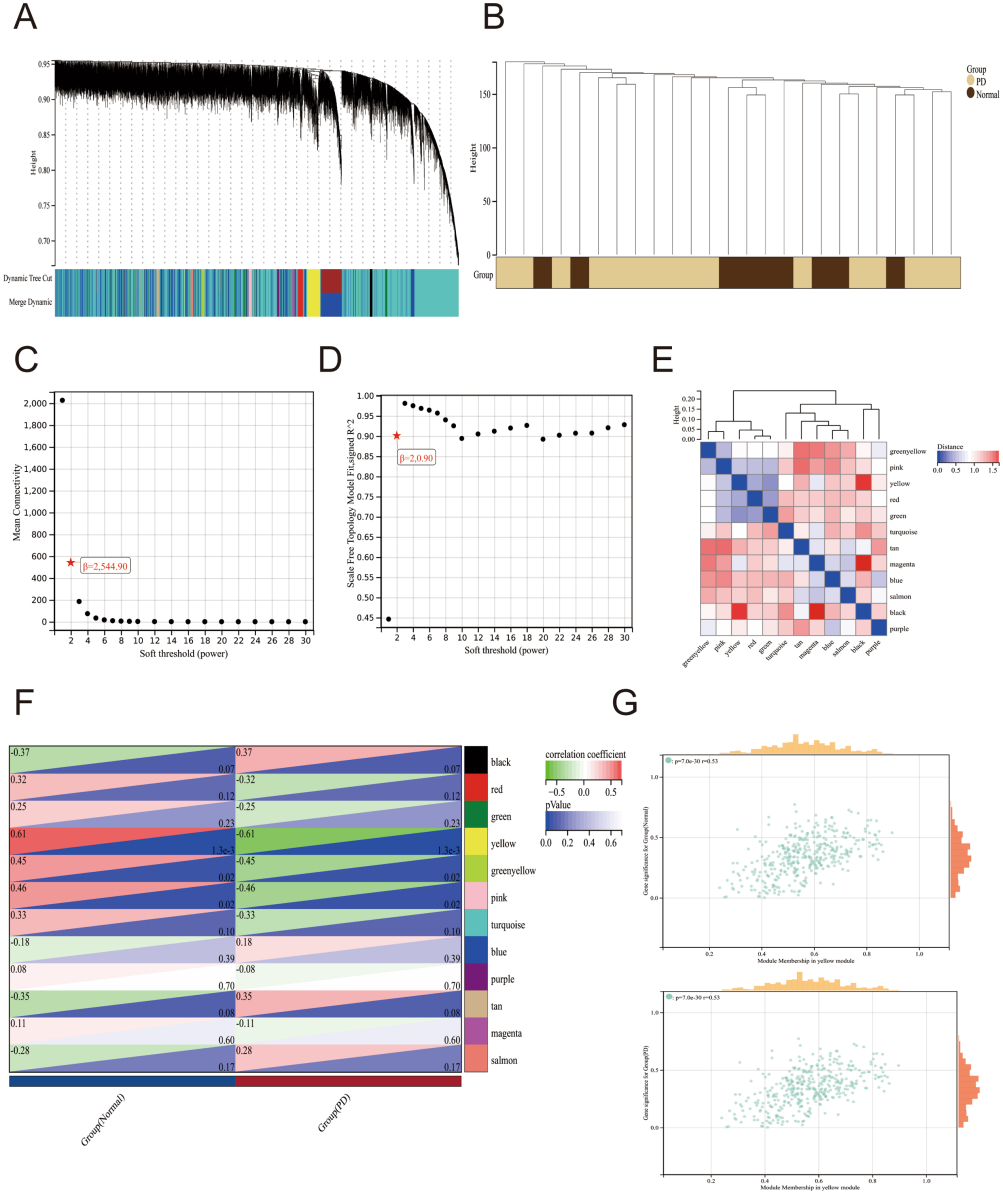
Identification of the correlated gene module for PD patients. **(A)** Dendrogram of genes clustered by WGCNA, with different colors representing distinct gene modules. **(B)** Dendrogram of samples clustered by the WGCNA, with different colors representing distinct gene modules. **(C,D)** Analysis of network topology to determine the soft-thresholding power. **(E)** Heatmap of eigengene adjacency illustrating the relationships among different gene modules. **(F)** Module trait relationship heatmap generated by the WGCNA in the internal set. **(G)** Scatterplots of representative modules associated with PD and control in the internal set.

Using WGCNA in the internal set, soft-thresholding powers of 2,544.90 and 2,0.90 were selected to achieve scale-free topology (Figures 3C,D). Hierarchical clustering of genes allowed the construction of multiple co-expression modules (Figures 3A,B), and their interrelationships were further visualized through a module clustering heatmap (Figure 3E). By correlating co-expression modules, we found that the yellow modules were most strongly associated with PD (Figures 3F,G).

### 3.3 Identification of druggable hub gene in the SN for PD patients

**Figure 4:**
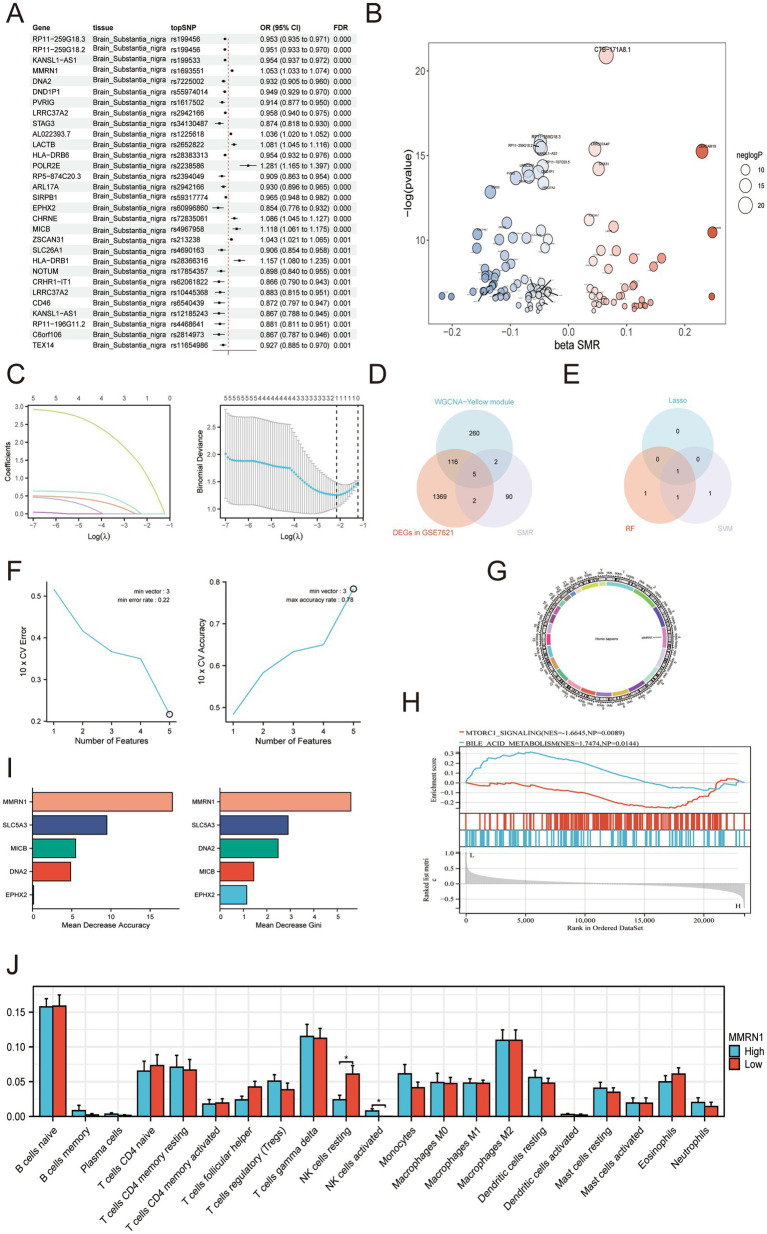
Identification of druggable hub indicator for PD patients. **(A)** Distinct druggable target for PD illustration of SMR results. **(B)** Rank of distinct druggable target for PD illustration of SMR results. **(C)** LASSO regression analysis for feature selection among candidate druggable targets. **(D)** Venn diagram illustrating the druggable DEGs. **(E)** Venn diagram illustrating the intersection of candidate genes identified by LASSO, RF, and SVM. **(F)** SVM curves showing the cross-validation accuracy and error rate across different feature subsets. **(G)** Chromatin localization of MMRN1. **(H)** Single-gene GSEA enrichment in the training set. **(I)** RF analysis ranking the importance of candidate druggable targets. **(J)** CIBERSORT-based immune cell infiltration analysis of MMRN1.

In accordance with the ebi-a-GCST90018894 dataset related to PD obtained from the IEU Open GWAS database and TwoSampleMR, the exposure variable was a druggable protein, and the outcome of interest was SN-related PD outcomes. A total of 99 druggable targets were identified, including 30 distinct targets (Figures 4A,B). Next, we intersected SMR results, Limma results, and WGCNA results, and we identified 5 druggable DEGs (Figure 4D). Next, based on these 5 DEGs, we applied LASSO, RF, and SVM for hub variable identification in the training set (Figures 4C,E,F,I). Integrative analysis of the three methods revealed one overlapping hub gene, MMRN1 (Figures 4E,G). To explore the molecular role of TAZ, we performed single-gene GSEA analysis. The results demonstrated that MMRN1 was significantly associated with signaling pathways, including mTORC1 and bile acid metabolism (Figure 4H). Furthermore, CIBERSORT immune cell infiltration analysis indicated that the higher expression of MMRN1 was correlated with the activation of NK cells (Figure 4J).

### 3.4 Novel diagnostic model construction in SN for PD patients

**Figure 5:**
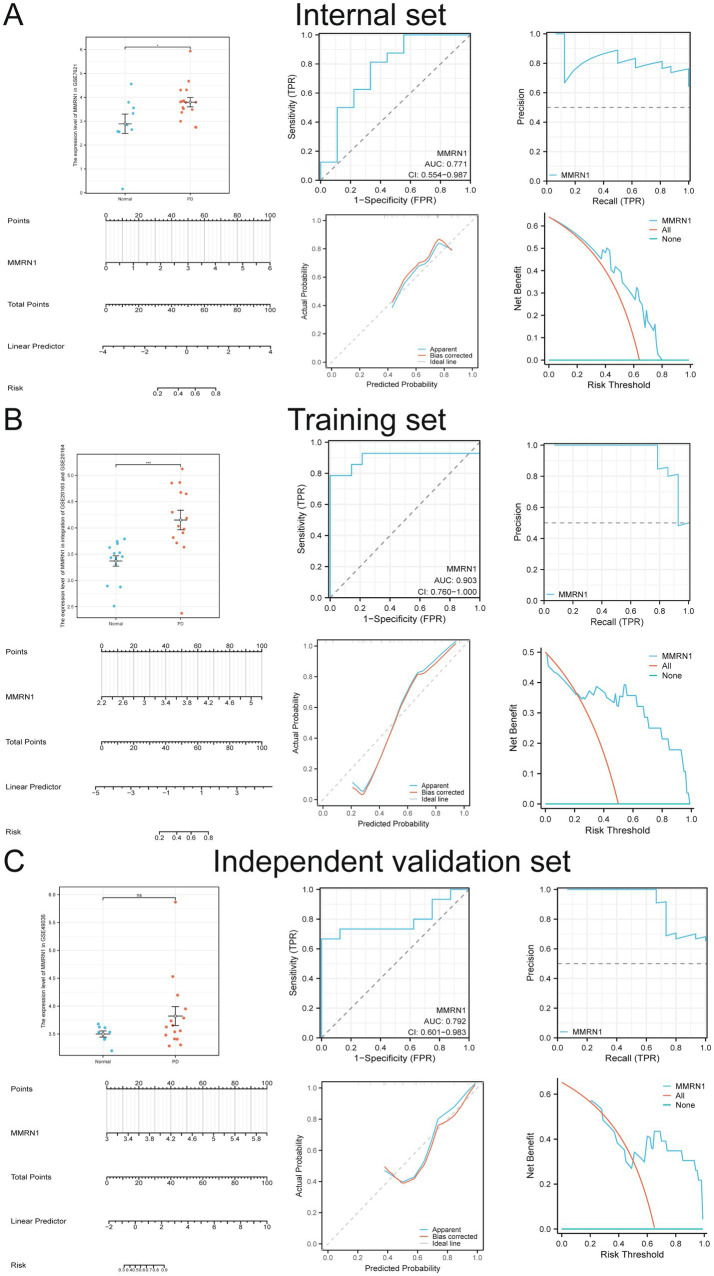
Cross-validation of diagnostic performance of MMRN1 in PD. **(A)** Expression and diagnostic value evaluation of MMRN1 in the internal set. **(B)** Expression and diagnostic value evaluation of MMRN1 in the training set. **(C)** Expression and diagnostic value evaluation of MMRN1 in the independent validation set.

We next validated the expression and diagnostic performance of MMRN1 for PD in the internal, training, and independent validation sets. Expression analysis revealed significantly higher MMRN1 expression levels in PD patients than healthy controls across all datasets (AUC = 0.771 in the internal set, AUC = 0.903 in the training set, and AUC = 0.792 in the independent validation set) (Figures 5A–C). To assess the diagnostic efficacy and accuracy of MMRN1, ROC, PR, DCA, nomogram, and calibration analyses were used across the internal, training, and independent validation sets. Collectively, the findings illustrate that MMRN1 serves as a robust biomarker with favorable diagnostic performance for PD (Figures 5A–C).

### 3.5 The single-cell landscape of MMRN1 in the SN for PD patients

**Figure 6:**
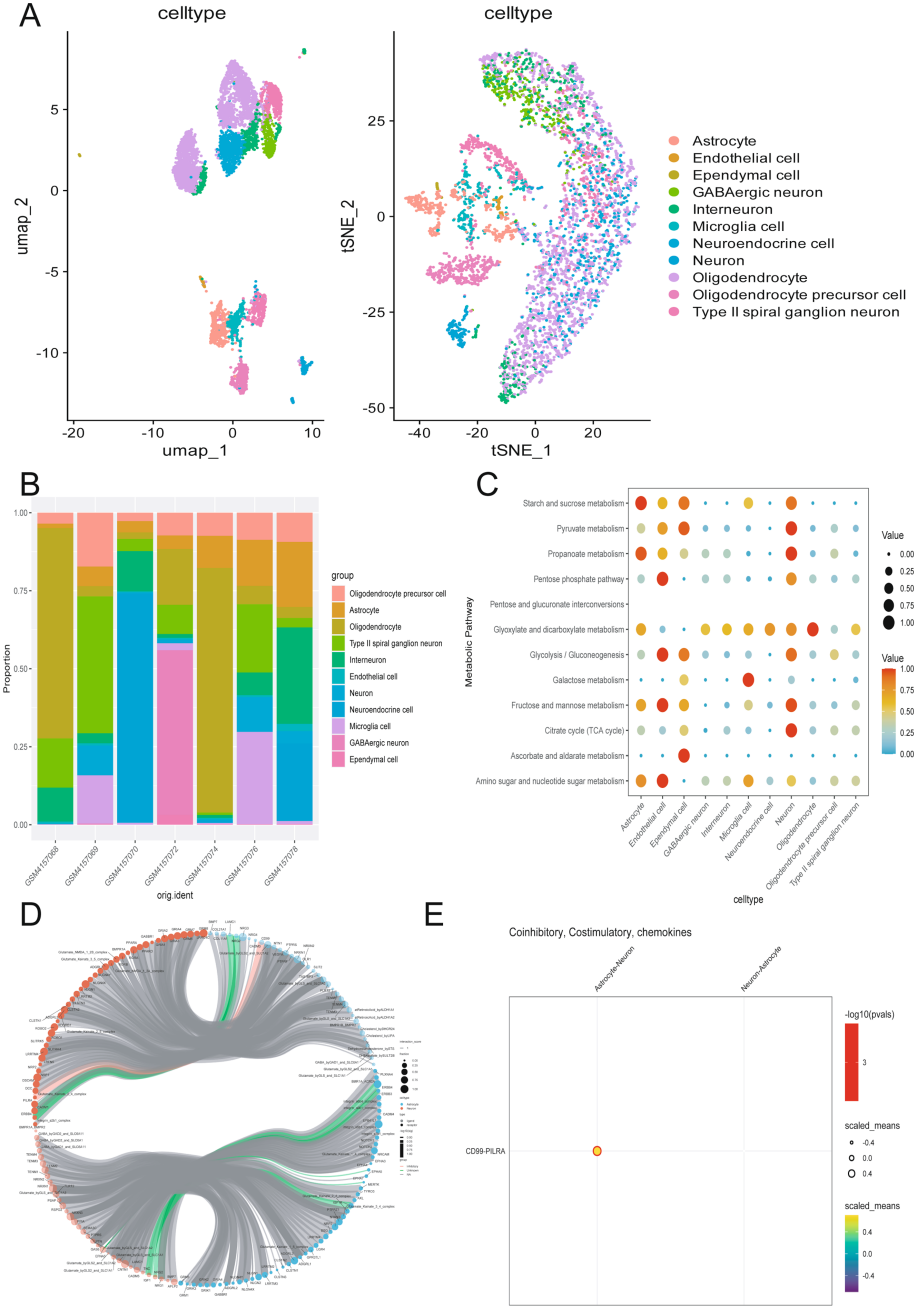
Global single-cell analysis of MMRN1 in the SN of PD patients. **(A)** UMAP and t-SNE plots displaying 11 annotated cell types. **(B)** Cell proportion annotated cell types. **(C)** Energy metabolism of 11 distinct cell types. **(D,E)** Metabolic heterogeneity among 11 annotated cell types.

After pre-processing of single-cell data (GSE140231), we identified 18 cell clusters and 11 cell types (Supplementary Figures S1A–E; Figures 6A,B). Next, cell energy metabolism enrichment was also performed (Figure 6C). In addition, the neuron primarily interacted with astrocytes via the CD99-PILRA axis (Figures 6D,E).

### 3.6 MMRN1 performance in SN neurons of PD patients

**Figure 7:**
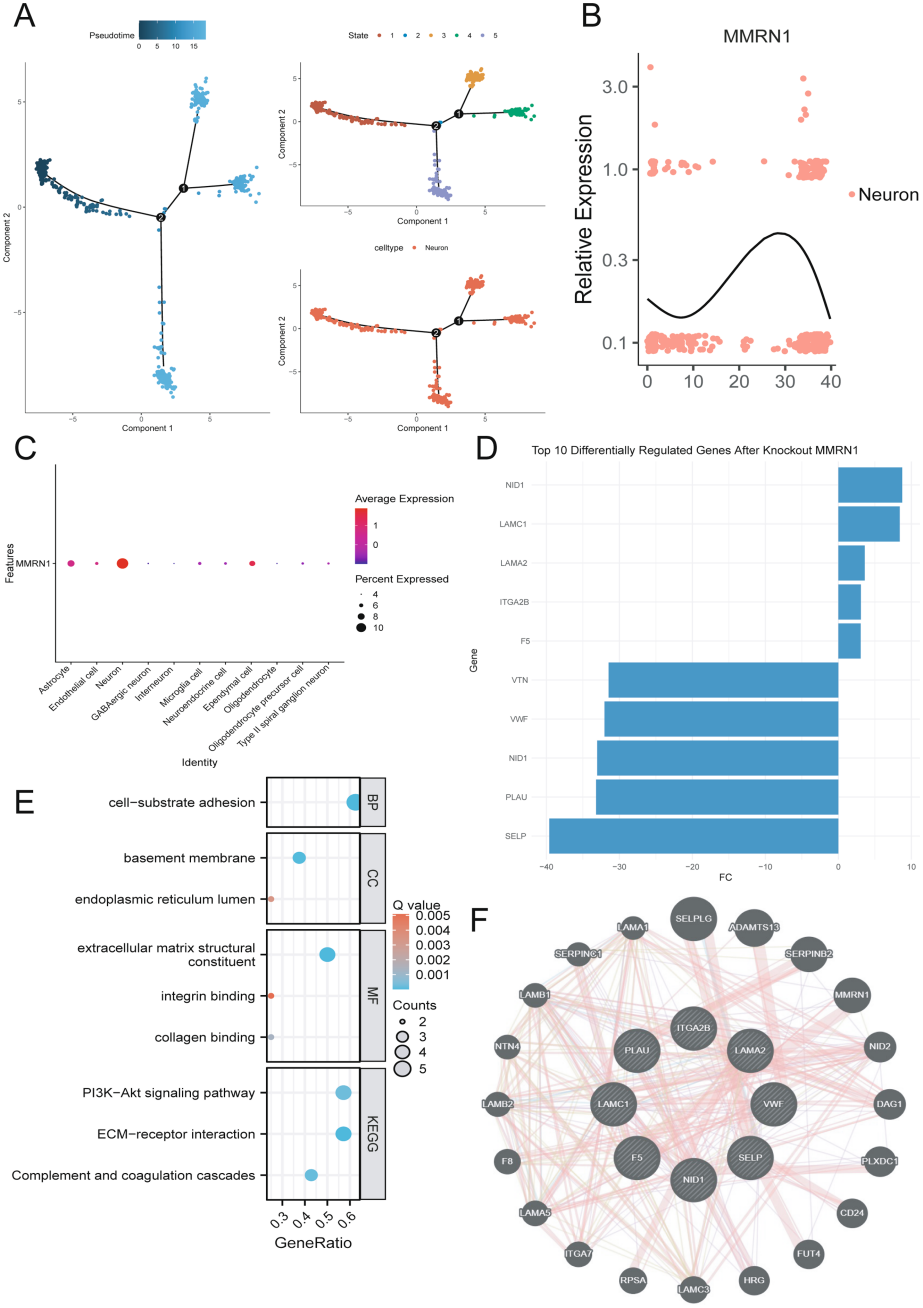
MMRN1 molecular insights into neurons. **(A,B)** Monocle 2 analysis of neuron and MMRN1. **(C)** Distribution of MMRN1 among annotated cell types. **(D–F)** Virtual KO of MMRN1 in neurons and corresponding molecular function estimation.

We first identified that MMRN1 was mainly distributed in neurons (Figure 7C). Next, we discovered that neurons illustrated five differentiation patterns and MMRN1 illustrated expression shifts during neuron differentiation (Figures 7A,B). Notably, virtual KO of MMRN1 in neurons can change the molecular patterns associated with neuroinflammation and blood–brain barrier maintenance (Figures 7D–F).

### 3.7 *In vitro* examination of MMRN1 expression and targeted therapeutic agent enrichment for PD patients

**Figure 8:**
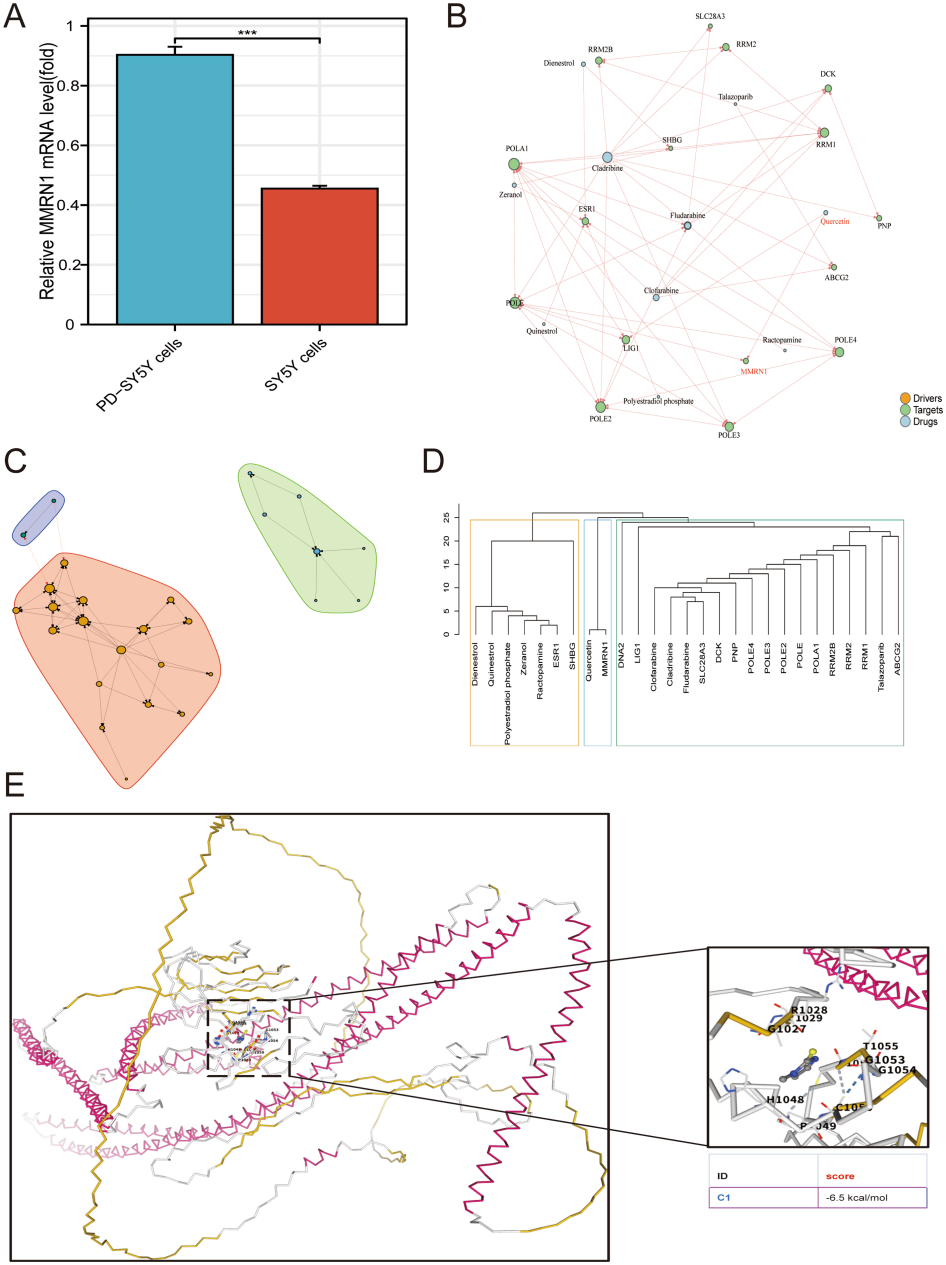
*In vitro* examination of MMRN1 expression patterns and targeted drug enrichment. **(A)** Expression and diagnostic value evaluation of MMRN1 in PD and non-PD cell lines. **(B–D)** Network-based *in silico* drug efficacy screening. **(E)** Molecular docking validation.

In MPP + SY5Y cells Compared with SY5Y cells, MMRN1 exhibited an upregulated expression pattern (Figure 8A). After network-based *in silico* drug efficacy screening, quercetin was identified as an optimal drug repurposing strategy for the treatment of PD (Figures 8B–D). In addition, the binding affinity between MMRN1 and quercetin was assessed, and the results indicated that quercetin has satisfactory binding affinity with the MMRN1 C1 pocket (Figure 8E).

## 4 Discussion and conclusion

Our integrative multi-omics analysis identifies MMRN1 as a central pathogenic factor in the SN of PD patients. While MMRN1 is best characterized as a platelet and endothelial cell adhesive glycoprotein involved in hemostasis and vascular integrity, its significant upregulation in PD neurons and strong diagnostic performance suggest a critical, neuron-intrinsic role in neurodegeneration (Laszlo et al., 2015; Leatherdale et al., 2021).

In addition to intracellular signaling, MMRN1’s known biology as an extracellular matrix (ECM) component allows us to hypothesize its role in ECM disruption and synaptic integrity (Wu et al., 2024). The brain ECM is crucial for synaptic stability, neuronal communication, and blood–brain barrier (BBB) maintenance (Pintér and Alpár, 2022). Pathological alterations in the ECM are increasingly recognized in neurodegenerative diseases (Pintér and Alpár, 2022; Freitas et al., 2021). The virtual knockout of MMRN1 in neurons significantly perturbed genes related to neuroinflammation and BBB maintenance in our single-cell analysis. This positions MMRN1 as a potential mediator connecting neuronal stress to the activation of astrocytes, thereby fueling a chronic neuroinflammatory milieu. Furthermore, by affecting perineuronal nets, MMRN1 dysregulation could compromise synaptic plasticity and resilience, accelerating circuit dysfunction in PD. In addition, for biomarker development, detecting MMRN1 in accessible biofluids is a logical next step. Although the primary source in PD may be neuronal, platelets or neuron-derived exosomes in the blood or cerebrospinal fluid could serve as surrogate carriers, providing a minimally invasive window into nigral pathology (Pinnell et al., 2021). However, assay sensitivity, specificity, and the correlation between peripheral and central nervous system levels require rigorous validation in large, prospective cohorts. Regarding therapy, our *in silico* screening and docking nominated the natural flavonoid quercetin as a high-affinity ligand for MMRN1. Quercetin possesses well-documented antioxidant, anti-inflammatory, and neuroprotective properties, making it an attractive candidate for PD (de Oliveira Vian et al., 2024). However, its clinical application is hindered by poor bioavailability and uncertain BBB penetration. Future strategies may involve the development of quercetin analogs with improved pharmacokinetic profiles, nanoparticle-based delivery systems, or its use as part of a combinatorial nutraceutical approach to enhance brain exposure and efficacy.

In conclusion, through a convergent bioinformatics and multi-omics pipeline, we have identified MMRN1 as a novel, neuron-enriched diagnostic biomarker and a plausible therapeutic target for PD. We propose a multi-faceted mechanistic model in which MMRN1 contributes to PD progression by potentially influencing the disruption of the extracellular matrix and exacerbating neuroinflammation. The associated diagnostic signature holds promise for patient stratification, while the nomination of quercetin provides a testable lead compound for therapeutic development. However, there are also further limitations that should be addressed. For example, MMRN1 diagnostic model construction for PD patients in our study was based on a public cohort, which lacks the real-world patient cohort for the examination of MMRN1 diagnostic model performance. Future studies should be focused on the validation of MMRN1 diagnostic efficacy and accuracy in a large and multi-center PD cohort to enhance the clinical translational potential of MMRN1. In addition, for MMRN1 molecular patterns in PD pathogenesis, our results were based on *in silico* enrichment, necessitating that future studies should validate our computational findings in pre-clinical studies. Furthermore, the quercetin protective effects for PD patients should be validated in pre-clinical and clinical trials to examine the corresponding efficacy and safety. In addition, quercetin pharmaceutical mechanisms by targeting MMRN1 should be estimated in future pre-clinical studies for increasing the transparency of the availability of quercetin in the treatment of PD.

## Data Availability

The original contributions presented in the study are included in the article/Supplementary material, further inquiries can be directed to the corresponding author.
