# Supplementary material for: Deciphering MMRN1 diagnostic and therapeutic implications in the substantia nigra of Parkinson’s disease patients via integrative bioinformatic analysis and multi-omics studies
Source: Front Aging Neurosci. 2026 Jun 18;18:1761327. doi: 10.3389/fnagi.2026.1761327 (PMC13323139; doi:10.3389/fnagi.2026.1761327)
Supplement: Supplementary file 1 [file Image_1.pdf]

# Supplementary Material

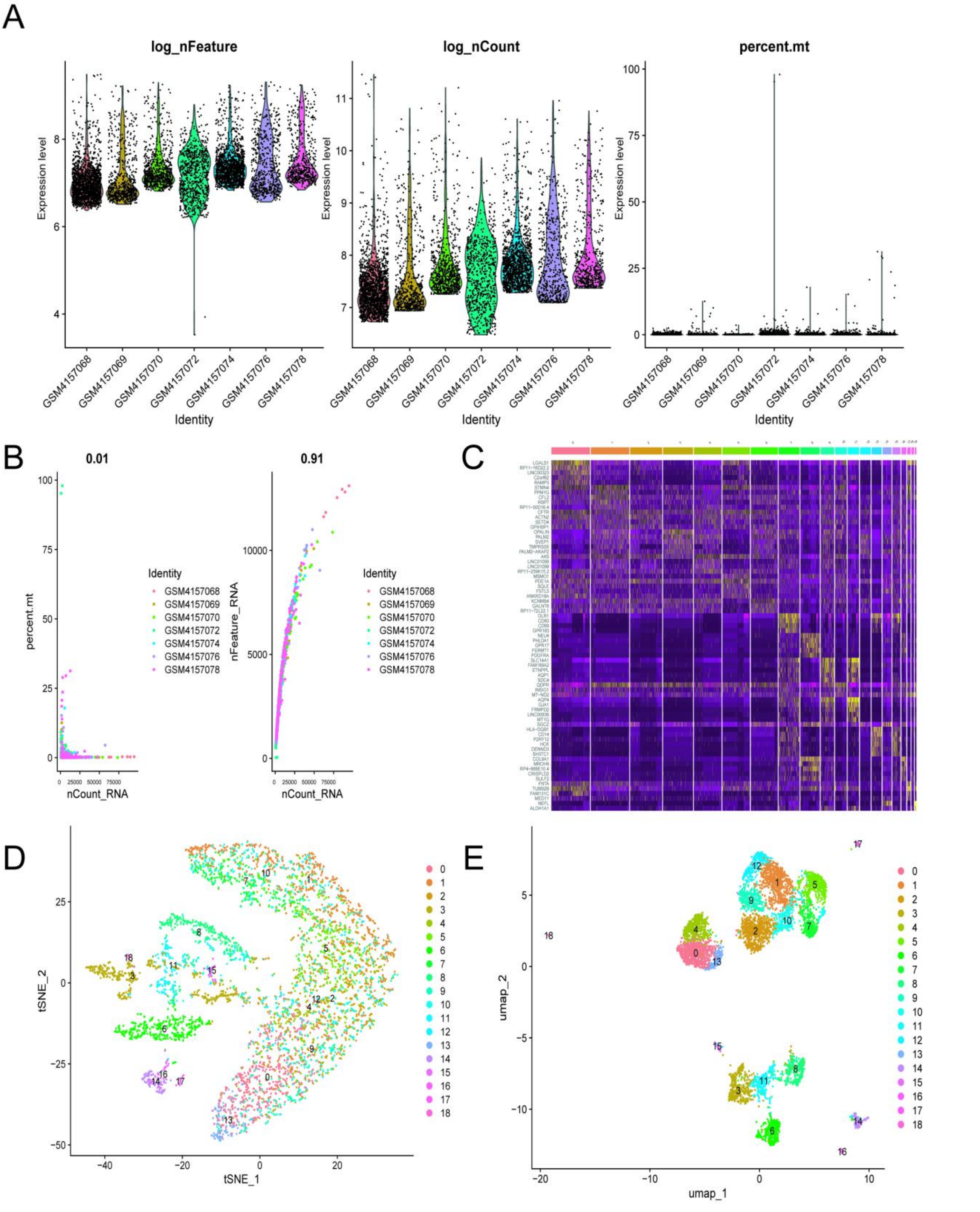


**Supplementary Figure S1.** Quality control and clustering analysis of single-cell RNA-seq data from the substantia nigra of Parkinson's disease patients. **(A)** Violin plots showing log_nFeature, log_nCount, and mitochondrial gene percentage across samples. **(B)** Scatter plots showing the relationships between nCount_RNA and percent.mt or nFeature_RNA. **(C)** Heatmap showing representative marker gene expression across cell clusters. **(D)** t-SNE visualization of cell clusters. **(E)** UMAP visualization of cell clusters.
